# Effect of Vibro-Tactile Stimulation Sequence and Support Surface Inclination on Gait and Balance Measures †

**DOI:** 10.3390/brainsci15020138

**Published:** 2025-01-30

**Authors:** Christopher P. Engsberg, Nathaniel H. Hunt, Steven Barlow, Mukul Mukherjee

**Affiliations:** 1 Department of Biomechanics, University of Nebraska at Omaha, Omaha, NE 68182, USA; cengsberg@unomaha.edu (C.P.E.); nhunt@unomaha.edu (N.H.H.); 2 Center for Brain, Biology, and Behavior, University of Nebraska Lincoln, Lincoln, NE 68588, USA; steven.barlow@unl.edu

**Keywords:** biomechanics, feedback, sensory, online, touch, haptics, walking, insole, skin, kinematics

## Abstract

The plantar surfaces of the feet are important for balance control during walking, specifically by allowing for the perception of pressure movements during stance. **Background/Objectives**: The current study aimed to perturb CoP movement perception in healthy individuals by applying vibrations to the soles of the feet in different stimulation sequences: a natural pattern that followed CoP movement (*gait-like*) and a perturbing pattern that did not follow the CoP (*random*) during walking. We hypothesized that the *gait-like* stimulation sequence would be similar to walking without any stimulation and therefore have no effect on balance measures and that the *random* sequence would negatively affect balance measures such as the anteroposterior (AP) and mediolateral (ML) margins of stability (MoSs) and foot placement area. **Methods**: Subjects walked at a level angle and 5.0 and 8.0 degrees of incline and with low visual conditions to increase reliance on tactile sensations from the feet. **Results**: No significant effect of the stimulation sequence was found at any incline, while there was a significant effect of incline. As the incline increased from level to 5 deg, subjects reduced their AP MoS measured at heel strikes from 4.36 ± 0.56 cm to 1.95 ± 1.07 cm and increased their foot placement area from 24.04 ± 11.13 cm^2^ to 38.98 ± 17.47 cm^2^. However, the AP MoS measured at midstance did not significantly change as the incline increased. **Conclusions**: The stimulation sequence had no effect on the dependent measures, but the subjects could still feel the vibrations on the plantar surfaces during walking; this implies that similar stimulation techniques could be a useful method for applying directive biofeedback without negatively impacting gait. Overall, this study demonstrates the detailed control of our tactile system and the adaptability of healthy individuals while walking with a perturbing stimulation.

## 1. Introduction

The cutaneous receptors within the plantar surfaces of the feet have been established as being important for balance control during standing and walking [1,2]. Specifically, during standing, they supply feedback about changes in pressure along the skin’s surfaces to keep the center of mass (CoM) over the base of support (BoS) [1,3]. This is performed through sensing the location and movements of the center of pressure (CoP) with respect to the CoM, aiding in the perception of the body’s orientation in space [3,4]. However, during walking, this CoP traverses along the foot in a repeated and predictable pattern, going from the heel to the toes along the lateral border of the sole [5]. The CoP is even adjusted during gait to keep the CoM within the BoS [6]. Thus, the perception of this CoP movement may be vital to the central nervous system (CNS) for keeping the CoM in balance over the base of support during walking.

A prominent symptom of hyperglycemia from diabetes is a loss of tactile sensation in the soles of the feet [7]. Due to this loss of sensation, people with diabetic neuropathy are at a far greater risk of falling than people with normal plantar sensitivity [7,8]. There has also been a significant relationship between the plantar sensitivity of the forefoot region and scores on clinical mobility measures [9]. This increased risk of falling and decreased mobility may be due to the lack of perception of tactile receptors along the foot’s sole. Interestingly, when only specific regions of the plantar surfaces are desensitized in healthy individuals, the majority of pressures are shifted away from desensitized regions and towards regions that remain sensitive to tactile stimuli [5]. This could demonstrate a preference and benefit for the CNS to effectively perceive tactile feedback along the foot’s sole while walking for appropriate balance control. Therefore, methods that influence or perturb the tactile receptors may negatively impact balance control.

One such method of influencing tactile perception could be using vibrations. Vibrations stimulate specific mechanoreceptors that are sensitive to a dynamic or moving stimulus [10], possibly like the movement of the CoP. During standing, vibrations applied to regions of the plantar surfaces led to individuals leaning away from such stimuli [3], with a higher frequency increasing this effect [11]. It was suggested by the authors that these high-frequency vibrations induced a perception change in the CoP location. Specifically, the feeling of the CoP shifted towards the locations of the vibrations. Thus, leaning away was a corrective measure to shift the CoP back to the original position [3,11].

The application of vibrations to the plantar surfaces during gait has been used previously to investigate its effect on spatiotemporal measures such as stride time and length [12,13,14], kinematic and kinetic measures [15,16,17], and variability measures [12,18,19,20]. To the best of our knowledge, there have been no studies investigating how moving vibro-tactile stimulation can alter gait (see review: [21]). Therefore, the purpose of the current study was to investigate if vibrations that moved along the plantar surfaces during walking could impact balance measures by perturbing plantar tactile receptors. This was performed by testing two different patterns of stimulation with healthy individuals during gait: a *gait-like* stimulation that followed CoP movement according to their real-time movements [22,23] and a *random* stimulation that was an unpredictable perturbing pattern. Both stimulation sequences only provided vibrations to the feet when the ground was perceived, during the stance phase of the respective foot. We hypothesized that the *gait-like* stimulation would supplement the natural sensation of pressure movements and thus not negatively impact balance measures whereas the *random* stimulation would negatively affect the perception of such movements and thus decrease balance measures. Alternatively, a lack of significant differences would indicate a healthy human ability to adjust and reweight, through multisensory integration and residual sensory feedback, such that gait and balance outcomes would show minimal or no deficits.

It is important to note that gait is controlled through multiple senses being integrated together; this is called the multisensory integration model [24,25,26]. Specifically, vision is one of the most relied-on senses for walking control [25,26], and removing vision during walking has been shown to produce increased brain activity in sensorimotor regions [27]. Thus, the current study had subjects walking with reduced visual information to increase their reliance on tactile input for balance control and possibly increase the effectiveness of tactile perturbation through the *random* stimulation pattern. Additionally, walking on an incline could further the reliance on the tactile system due to the increased risk of a slip [28]. For these reasons, subjects walked in low light conditions at three different inclines, leading us to form an additional hypothesis that the effect of the *random* stimulation would increase as the walking incline increased.

**Figure 1:**
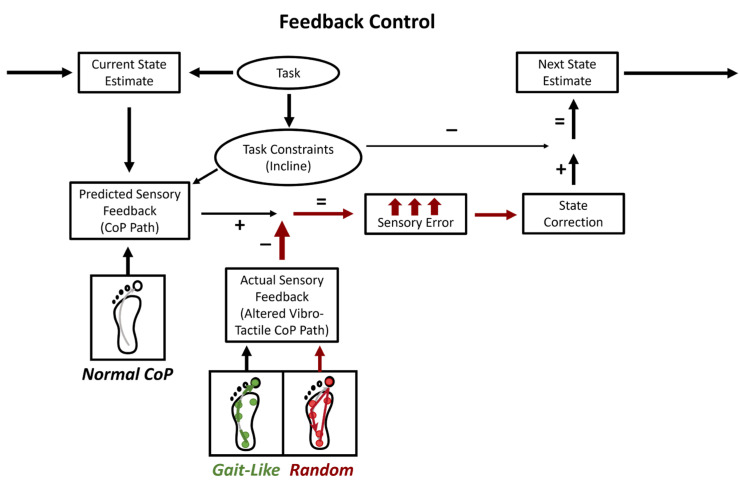
Sensorimotor control requires the comparison of an internal model of the current state to the actual state to generate corrections for reaching a desired state. Task constraints are influenced by the specific task, to shape the corrections, and the expected sensory feedback. In this experiment, the treadmill incline altered task constraints, and the plantar stimulation patterns may have affected how the actual sensory feedback aligned with the predicted feedback (e.g., the natural CoP path) to maintain balance during walking. We hypothesized that increased sensory error from unexpected stimulation (random pattern) may lead to greater state corrections and, potentially, balance deficits. Red arrows depict where *random* stimulation alters model outputs. Conversely, effective corrections without deficits would suggest a healthy system. Gait-like stimulation was expected to align with the natural CoP path, not affecting sensory error.

If our assumptions were correct, this experiment would allow us to test our model and how the CNS uses tactile information for walking with a feedback control system (Figure 1). This model was tested by altering the actual sensory feedback, with the *gait-like* and *random* stimulation sequences, to compare it with the predicted sensory feedback of normal walking.

## 2. Materials and Methods

### 2.1. Subjects

For this study, a total of 14 healthy adults (7 male, 7 female; age: 26.9 ± 3.1; height: 167.6 ± 8.8 cm; weight: 71.1 ± 20.2 kg) were recruited. These individuals were between the ages of 19 and 30 years of age, with exclusion criteria being the presence of any dysfunction including physical impairments, neurological disease, cardiovascular disease, or other abnormalities that may affect walking on a treadmill. Each participant gave informed consent before their participation. Ethical approval was provided by the institutional review board from the University of Nebraska Medical Center (0228-22-FB).

### 2.2. Equipment

Prior to walking, all participants performed a set of pre-tests to determine plantar sensitivity. This included Semmes–Weinstein monofilaments (North Coast Medical Inc., Morgan Hill, CA, USA) and a 120 Hz biothesiometer (Bio-Medical Instrument Company, Newbury, OH, USA). These tests were performed to inspect perception sensitivity to pressure and vibrations on the plantar surfaces.

**Figure 2:**
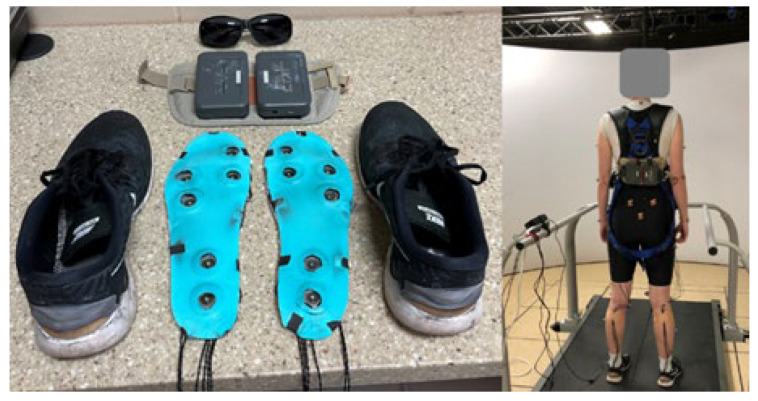
The experiment and equipment set-up for data collection. (**Left**) Custom-made tactor-embedded insoles that fit into the specific shoe size of each subject. These tactors were then connected to the tactor boxes that were attached to a fanny pack using Velcro. Subjects wore sunglasses in the dark room to decrease visual information and increase reliance on tactile feedback. (**Right**) Subjects wore the fanny pack around their waist such that the tactor boxes were on their back above the posterior pelvic markers.

The subjects were then given a pair of Nike Free minimalist shoes with custom-made tactor-embedded insoles (Figure 2). Each insole was fitted with six C-2 tactors (Engineering Acoustics Inc.; EAI, Casselberry, FL, USA), placed in sets of two under the heel (heel set), the base of the fifth metatarsal (MT5 set), and the base of the first metatarsal and big toe (MT1 set). These tactors were set to vibrate at a constant frequency of 250 Hz [12,13] and maximum amplitude of 23.5 db (~0.2 mm). This frequency and amplitude combination with the C-2 tactors has been shown to be perceivable during standing and walking in healthy and patient populations [12,13,18]. The participants walked on a force plate-instrumented split-belt treadmill (Bertec Version 2.0 2013, Columbus, OH, USA) that collected ground reaction forces (GRFs) at 1000 Hz. Marker position data were collected using a 16-camera motion capture system (Vicon, Oxford, UK) at 100 Hz. Reflective markers were placed on bony landmarks following the PlugInGait Full-Body AI from Vicon (Figure 2). Two tactor controlling boxes, from EAI, were attached to the lower back of the subjects via a fanny pack such that there was one box for each shoe. These boxes were controlled through custom-made MATLAB software (version R2024a) [23].

**Figure 3:**
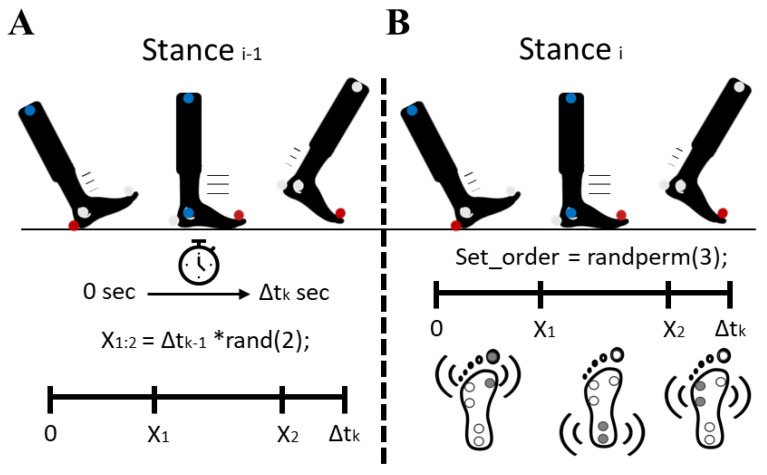
(**A**) The stance time of the previous step was used to determine two random time points (X_1_ and X_2_) within the time of heel-strike-to-toe-off (Δt_k_). Multiplication sign is denoted with “*”. (**B**) During the current stance, a random order of tactor sets was calculated through performing a random permutation from 1 to 3. Then, once the foot had been in the stance for X_1_sec, the first tactor set changed to the second set; then, reaching X_2_sec led to the final tactor set being active until toe-off occurred.

This MATLAB controller allowed for the real-time control of individual tactor sets within each shoe to give stimulation according to the different phases of gait based on real-time kinematic data. With such control of the stimulation, the subjects walked while experiencing three different stimulation sequences: *no stimulation*, *gait-like* stimulation, and *random* stimulation. This real-time controller was validated previously by our group for the *gait-like* stimulation [23]. This *gait-like* stimulation activated each set of tactors within one insole sequentially from the heel set during heel-strike-to-midstance, the MT5 set during midstance-to-heel-lift, and the MT1 set during heel-lift-to-toe-off. This was meant to follow the progression of the normal CoP progression along the plantar surface during walking (Figure 3). The *random stimulation* caused a random sequence of the three sets to be activated sequentially for random durations during the stance phase of gait. This meant that the tactor sets activated in a perturbing pattern, against the normal CoP progression and not according to the real-time movements. Finally, *no stimulation* was treated as a control condition, with no tactors being activated during stance.

### 2.3. Procedure

After the monofilament and biothesiometer plantar sensory tests, the subjects performed a tactor vibration familiarization test and a treadmill walking familiarization trial. The first was familiarization to the feeling of the vibrating tactors on different locations of the feet. This was performed to remove any surprise effects from when the tactors were first activated during the walking trials and to be sure that each participant could locally feel each individual tactor set. The subjects stood while wearing the tactor insoles as each tactor set between the two feet were activated. The subjects were asked to state which tactor set was activated using a key showing the tactor set locations (Supplementary Figure S1). The second familiarization trial was to become comfortable with the walking conditions. For this, the subjects performed a 5 min walk to habituate to the conditions of low light and sunglasses [29,30,31,32] during level walking at 0.8 m/s with no stimulation.

**Figure 4:**
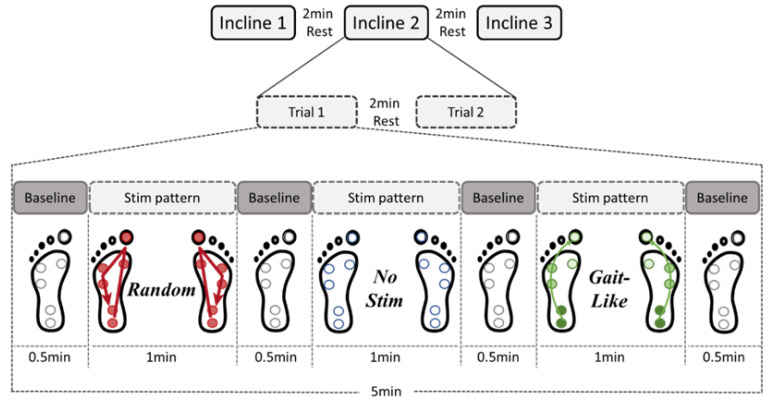
The protocol of an example trial. The order of stimulation patterns was randomized between trials for each of the three inclines. Individuals walked for a total of 5 min where 1 min of each stimulation pattern was experienced. There were 30 s breaks with no stimulation between the three patterns to allow the subject to return to a normal baseline of walking before the next stimulation.

For the experimental trials, the participants performed two trials with each incline at 0.8 m/s [33], with the order of the inclines being randomized, with a break of at least 2 min between trials. This resulted in a total of six trials and 35 min of walking. Each trial consisted of 5 min of walking; within each trial, the subjects experienced 1 min of each stimulation sequence in a randomized order. These stimulation sections of the trial were separated by 30 s of NS (baseline) before and after each sequence, which was treated as a period for the subject to return to a baseline walking pattern before the next sequence began (Figure 4). Therefore, there were two minutes of walking (at least 90 steps) recorded for each stimulation sequence at each of the three inclines.

After the subjects completed all trials, we wanted to ensure that any gait changes we may have seen could not be attributed to pain or discomfort from the vibrations or the presence of the tactor-embedded insoles [34]. For this, we had the subjects provide a visual analog scale (0–10) rating of the general comfort of the insoles, with 0 being the least and 10 being the most comfortable. Then, we asked if that rating changed when the tactors were active compared to inactive. Finally, the subjects gave feedback on how they perceived the sequence of vibrations they experienced during the trials. The subjects were not told before participating about the different stimulation sequences; thus, this was a test of how attentive individuals were in sensing a moving vibratory stimulus on the plantar surfaces during walking. This was performed by asking if they felt anything different between the two moments of vibration during the walking trial at the end of the final trial.

### 2.4. Data Analysis

Data analysis was performed in MATLAB. Due to individuals walking with low visual information, step-overs onto the contralateral belt/force plate were common. For this reason, gait events were found using the velocity of the heel and toe markers of each foot. A heel strike was defined as a heel anteroposterior (AP) velocity change from positive (forward) to negative (backward), and toe-offs were detected as the toe marker velocity changing from negative to positive [35]. Spatiotemporal measures such as stance time, stance length, and stride width were calculated from these gait events. Stance time was the duration from a heel strike to the following toe-off on the ipsilateral side, and stance length was the limb excursion of the foot across the belt [36] normalized to the body height of each subject. Stride width was the mediolateral (ML) distance between the heel markers of both feet at each heel strike.

Foot placement was the mediolateral and anteroposterior position of the heel marker at heel strike with respect to the position of the center of mass (CoM). Foot placements were then analyzed further with a 95% confidence ellipse, created using Equations (1)–(3). This ellipse was made by finding the largest and smallest eigenvectors, depicted as $\lambda_{1}$ and $\lambda_{2}$, respectively, that described the locations of each foot placement for the duration of a stimulation sequence (1 min). These eigenvectors and values made up the direction and ratio of the major and minor axes for the ellipse. The angle of the ellipse, represented as ϕ, was calculated by finding the angle between the largest eigenvector and the x-axis. The radii of the ellipse were found by multiplying the square root of the eigenvalues by the Chi square value of 2.4477 that represented a 95% confidence interval. Then, the major ($r_{1}$) and minor ($r_{2}$) radii were multiplied by a two-dimensional rotation matrix based on the angles of the eigenvectors with respect to the x-axis (R(ϕ)). Thus, this resulted in a 95% confidence ellipse that was oriented according to the spread of heel strikes. The areas of these ellipses were compared between conditions (Supplementary Figure S2). A larger area represented more sporadic and widespread foot placements, and a small area represented more consistent foot placements with respect to the CoM.

(1) $$\phi =arctan\left(\frac{\lambda_{1}}{\lambda_{2}}\right)$$

(2) $$r_{1}=\left(2.4477\sqrt{\lambda_{1}}\right)\;\times \;R(\phi )\;r_{2}=\left(2.4477\sqrt{\lambda_{2}}\right)\times \;R(\phi )$$

(3) $$Area={\pi (r}_{1}\;\times \;r_{2})$$

The margins of stability (MoSs) were analyzed using the marker position and velocity data. The MoS was calculated as the minimum distance between the base of support (BoS) and the extrapolated center of mass (XCoM) (Supplementary Figure S3). The BoS was estimated as the position of the ankle marker [37], while the XCoM was calculated as described in Equations (4)–(6). In brief, it was the position of the CoM plus the velocity of the CoM (*v_com_*), including the walking speed for the AP direction, divided by the pendulum eigen frequency (ω_o_) [6]. The eigen frequency was calculated as the square root of the force of gravity (*g*) divided by the effective height of the CoM (*h*), which was 1.34 times the leg length (*l*) [6]. The position of the CoM was estimated by the average position of all the pelvic markers (ASISs, PSISs, and Sacrum).

(4) $$XCOM=x_{CoM}+\frac{v_{CoM}}{\omega_{CoM}}$$

(5) $$\omega_{CoM}=\sqrt{g/h}$$

(6) h=(1.34)l

The MoS was found in the ML and AP directions at different time points in the gait cycle. The ML MoS was calculated as the minimum distance between the XCoM and BoS throughout the stance phase of gait [6,37]. The AP MoS was found at the moment of heel strike and midstance [38,39,40,41]. Midstance was defined as the moment of the ankle marker becoming in line with the CoM.

### 2.5. Statistics

Statistical analysis was performed in SPSS 16.0 (IBM Corporation, Armond, NY, USA). To test significant differences between the effects of inclines and stimulation sequences, a 2-way 3 × 3 repeated measures ANOVA (Level/5Incline/8Incline x NS/GS/RS) was performed with a significance level of 0.05. A Greenhouse–Geisser correction was used if the data did not pass the sphericity assumption. If significant differences were found, a Tukey post hoc test was performed for finding the directionality of the differences. Unfortunately, due to tactor connection issues that were discovered after data collection, the data of 5 subjects had to be removed from analysis. This was determined through spectral analysis of the vertical ground reaction force and seeing a lack of a spectral peak at the 250 Hz range, caused by the tactor vibrations (Supplementary Figure S4). Thus, all analysis reported is of the remaining 9 subjects (4 male, 5 female; age: 26.2 ± 3.5; height: 166.7 ± 7.8 cm; weight: 62.5 ± 11.4 kg).

## 3. Results

Some subjects were removed from the final analysis. One subject was unable to complete all the trials, and during analysis it was discovered that the tactile system disconnected half-way through some trials for four of the participants. This was determined through spectral analysis of the vertical GRF and noting a lack of a spectral peak at 250 Hz in those trials, caused by the tactor vibrations (Supplementary Figure S4). These subjects were not used in the final analysis; thus, the results presented are from the remaining nine subjects (four male, five female; age: 26.2 ± 3.5; height: 166.7 ± 7.8 cm; weight: 62.5 ± 11.4 kg).

### 3.1. Sensory Perception

The sensory thresholds of the subjects were within normal ranges [42], with the monofilament test averaging around a size 4 filament (<1.4 g of force) and vibration perception within a 0.05 microns amplitude at 120 Hz (Table 1 and Table 2).

### 3.2. Spatiotemporal

**Figure 5:**
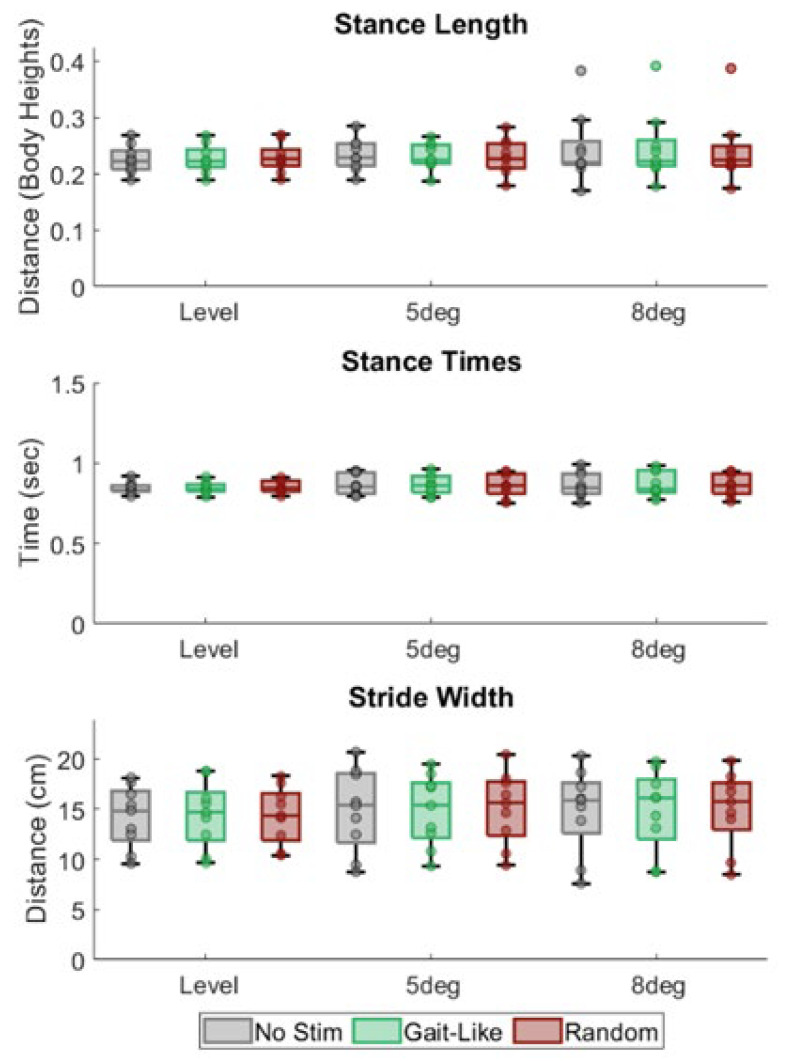
Box and whisker plots of quartiles for spatiotemporal results. Stimulation pattern and incline had no significant effect.

There were no significant main or interaction effects from the incline and stimulation sequence (Figure 5) on the stance time (incline: F = 1.148; *p* = 0.322; stim sequence: F = 0.159; *p* = 0.854), stance length (incline: F = 0.676; *p* = 0.440; stim sequence: F = 0.14; *p* = 0.87), or stride width (incline: F = 0.54; *p* = 0.593; stim sequence: F = 0.678; *p* = 0.522).

### 3.3. Balance Measures

**Figure 6:**
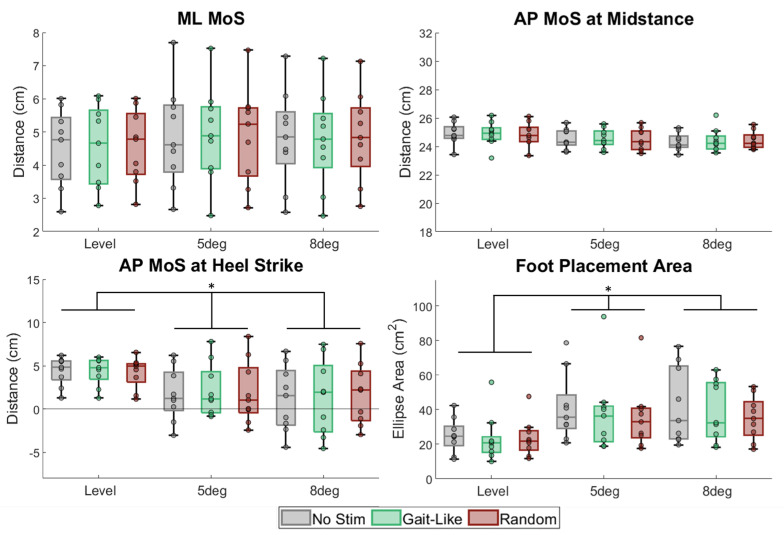
Box and whisker plots of quartiles for balance measures. Stimulation had no effect on balance measures. Incline had significant changes in foot placement area and AP MoS at heel strike. (* indicates *p* < 0.05).

There was no main effect of the stimulation sequence on any of the balance measures (Figure 6), the ML MoS (F = 0.709; *p* = 0.507), the AP MoS at heel strike (F = 0.609; *p* = 0.556), the AP MoS at midstance (F = 0.104; *p* = 0.902), and the foot placement area (F = 1.551; *p* = 0.242). However, there was a main effect of the incline on the AP MoS measures at heel strike (F = 6.503; *p* = 0.030) and foot placement area (F = 7.849; *p* = 0.004). As the incline increased from level walking, the foot placement area increased by about 14 cm^2^; however, there was no further increase as the incline increased from 5 to 8 deg. For the AP MoS at heel strike, walking at 5 and 8 deg of incline was significantly reduced by 2.41 cm and 2.96 cm, respectively. However, there was no difference in the AP MoS at heel strike when walking at 5 deg or 8 deg of incline. However, there was no main effect of the incline on the ML MoS (F = 1.46; *p* = 0.264) or AP MoS at midstance (F = 3.633; *p* = 0.088).

### 3.4. Comfort and Sequence Perception

Concerning the comfort of the tactors when they were inactive, it was reported as being average (5.89 ± 1.62). Similarly, when the tactors were active, the comfort was reported as average (5.67 ± 1.30). Some individuals seemed to have a preference for the tactors being ON or OFF (Subject 7 strongly preferred the tactors to be OFF, while Subject 5 preferred the tactors to be ON); however, these differences were averaged out when analyzing all participants (Table 3). Only two participants seemed to notice a difference in the activation sequences of the tactors, while the remaining participants perceived no differences between the different stimulation sequences or thought that the strength of the vibrations was the difference (Table 4). All results can be seen in Supplementary Table S1.

## 4. Discussion

In the current study, we investigated the effects of different patterns of vibro-tactile stimulation on the plantar surfaces during the stance phases of gait. This was performed at different walking inclines and with low visual information to increase reliance on the tactile system. We found a lack of significant effects from stimulation patterns in both spatiotemporal and balance measures; only the incline led to a change in the AP balance measures. This goes against our original hypotheses that the *random* stimulation would negatively affect balance measures. Instead, it supports the hypothesis that healthy humans have the ability to adjust and reweigh, through multisensory integration and residual sensory feedback, such that gait and balance outcomes show minimal or no deficits.

### 4.1. The Effect of Incline on Balance Measures

**Figure 7:**
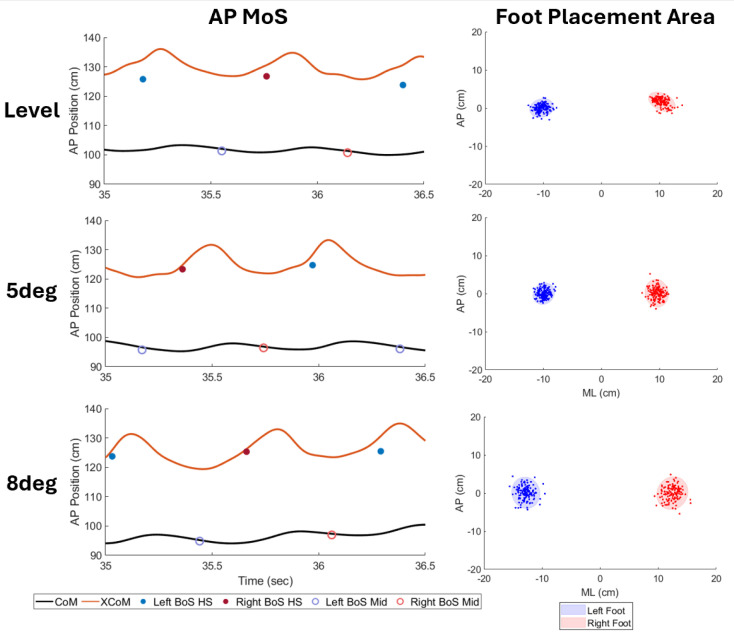
A representative subject of AP MoS changes and foot placement area changes over the levels of inclines. (**Left**) The AP MoS at heel strike reduced with incline and was the distance from the base of support at heel strike (filled circles) to the XCoM. The AP MoS at midstance was the distance from the base of support at midstance (open circles) to the XCoM. (**Right**) Foot placement area was the ellipse area for the two feet and increased with incline.

Incline influenced the MoS in the AP direction during heel strikes (Figure 6) but not in the ML direction. The AP MoS at heel strike reduced due to walking on the incline (Figure 7). Firstly, the decrease in the AP MoS at heel strike as incline increases has been shown previously [38,39]. Previous studies have determined changes in step length to be linked with these changes in the AP MoS during inclined and declined walking [38,39]. However, the current study found no significant changes in spatiotemporal measures, including step length (Supplementary Figure S5). Interestingly, further analysis of CoM velocity found that it increased during inclined walking, which would be expected to increase, not decrease, the AP MoS at heel strike. Therefore, further investigations of the AP MoS during inclined walking should be performed.

Concerning the ML MoS, previous studies have shown an increase during inclined walking [38,39]. However, these studies have had individuals walk at their preferred walking speed, as opposed to the set slow walking speed in this study. Walking at a slow pace increases the ML MoS [43], and putting an individual in unstable walking scenarios also increases the MoS [44]. Thus, the lack of an ML MoS effect may have been from individuals already walking with an increased MoS.

### 4.2. Vibro-Tactile Stimulation in the Gait-like and Random Sequences Did Not Alter Gait Measures

Different stimulation sequences led to no significant changes in any of the variables tested in this study. These results contradict a previous study investigating the effects of sub- and supra-threshold plantar stimulation on stride time during different walking inclines [21]. Where the current study differs is the timing and sequences of stimulation. Most vibro-tactile stimulation studies have the vibrations present throughout the entire gait cycle, including the swing phase [12,13,21]. The timing of stimulation used in the present study occurred only during the stance phase of gait, when the plantar surfaces supply information about the environment. Interestingly, unlike the hand, the mechanoreceptors within the plantar surfaces do not have any passive activations with the absence of pressures, such as the swing phase of gait [45]. Thus, if the plantar surfaces are being stimulated with vibrations during swing, there would not be an augmentation of sensory feedback but a new, possibly perturbing, sensation.

It is possible that the lack of tactile stimulation effects could be due to healthy individuals being very adaptable to various walking conditions [24,25]. Therefore, there was the possibility that an effect was present when the stimulation exposure was initiated and stopped but was then averaged out from the subjects returning to a steady walking state. However, when analyzing the changes in these dependent measures in the steps just prior to and after (four steps on each side) the beginning of each stimulation sequence, and vice versa, no observable changes were noticed (Supplementary Figure S6).

We suggest that the current study shows that applying vibrations to the plantar surfaces in a true augmenting manner results in minimal effects to such gait measures. One reason for this could be that feeling the tactile stimulation was not paired with a particular purpose for walking. Thus, vibrations applied to the plantar surfaces could be used as a directive for a desired action through previous instruction. This has been performed previously to improve the gait symmetry of stroke survivors by applying vibrations to the calf [46]. Future studies could investigate such effects further.

### 4.3. Vibro-Tactile Stimulation May Not Affect the Perception of Pressure

In the current study, we attempted to alter the perception of pressure movements under the foot like postural studies that applied high-frequency plantar vibrations [3,11]. These studies found whole-body shifts away from vibro-tactile stimulation applied to the plantar surfaces, possibly due to a perceived shift in the CoP from the mechanoreceptor stimulation. However, in the present study, similar vibrations had no effect on gait measures. There are two main possibilities: (1) the plantar surfaces are not involved in the perception of CoP movement during gait or (2) vibrations do not alter the perception of the CoP.

It is possible that the time delay from tactile stimulation to its perception in the brain or spinal cord is too long for reliable balance control. By the time the brain learns how or where the CoP is moving, the system may already be in the next step. However, it has been shown that the CoP is shifted medially or laterally during the stance phase to maintain a stable ML MoS [6]. The plantar surfaces would be a great source of afferent feedback about the location of the CoP during these adjustments for a complete feedback control loop [47]. Further support comes from studies investigating the effects of decreasing plantar surface cutaneous sensitivity. This leads to changes in responses to perturbations during standing in both muscle activations [48,49] and emergent postural responses [50,51,52], as well as small immediate effects on the MoS during perturbed walking [53] and CoP shifts away from regions of desensitization [5]. Thus, the current study may specifically show that the addition of different vibration stimulation sequences does not influence the effectiveness of the used sensory information from the plantar surfaces.

Additionally, there have been studies that show a strong neural connection between the motor control centers of the brain and the sensory representations of the foot. There have been studies that show the activation of motor control centers, such as the supplementary motor cortex, just by stimulating the plantar surfaces in a gait-like sequence [54,55]. These studies suggest that these coactivations of sensory and motor areas are evidence of plantar tactile feedback being used in gait control. Therefore, while there was no effect of plantar stimulation found in the current study, it may not necessarily mean that the plantar surfaces were not being used for balance and gait control. It is possible that in the sufficiently long stance phase, multisensory integration allows adjustments of sensory weights such that balance and gait outcomes show minimal effects.

Therefore, it could be that the vibrations we supplied to the plantar surfaces did not alter the perception of the CoP and thus did not alter the gait measures tested. This could indicate that the healthy individuals were able to distinguish the sense of pressures applied to the foot from the ground and the vibrations supplied by the tactors. Fast-adapting (FA) fibers are most sensitive to vibrations and moving stimuli across the surface of the skin [10]. This led us to believe that these FA fibers may aid in perceiving the moving CoP during stance and thus that the addition of vibrations would negatively impact the signal-to-noise ratio and result in gait-related effects. However, SA fibers are known to feel pressure and give information on the level of pressures applied to the skin while not being sensitive to vibrations [56,57]. The rate of action potentials sent by these SA fibers reflect the amount of pressure applied to the skin [56]. Therefore, different regions of the foot supply a higher rate of action potentials at different moments of the stance phase. Healthy individuals may be able to make do with this reduced signal-to-noise ratio from FA fibers during stimulation because it may not be the main sensory fiber type that the CNS is using to sense the CoP movement.

**Figure 8:**
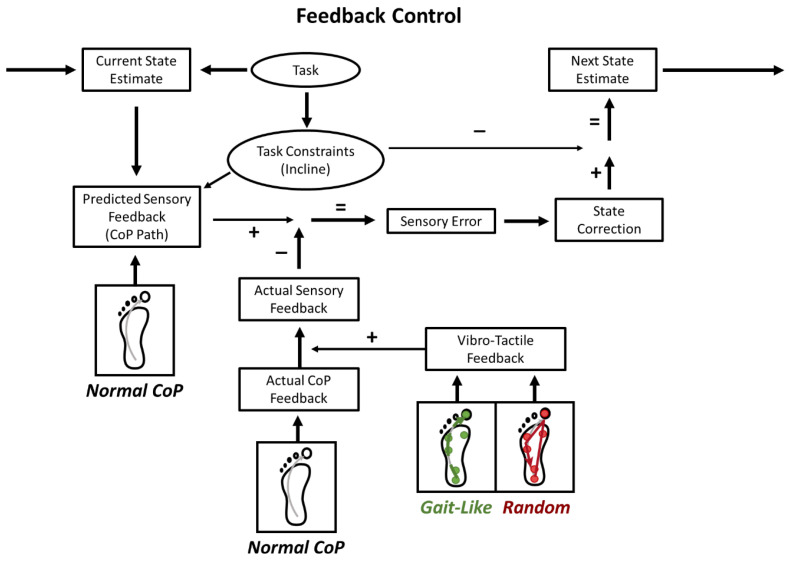
The feedback model revisited with the possible effects of stimulation. During abnormal stimulation, the CNS may perceive natural CoP movements and vibro-tactile patterns as distinctive inputs. Thus, most sensory feedback from the plantar surface consists of expected natural feedback, while additional vibro-tactile input is ignored or treated as irrelevant. Previously, we assumed that these inputs would intertwine, but in healthy individuals, even in slightly unstable walking conditions, this does not seem to be the case.

Additionally, the addition of vibrations altering the signal-to-noise ratio for sensing the CoP may only lead to changes in behavior if the task allows it. During standing, individuals can adjust the CoP throughout the BoS. However, during walking, the CoP movement is a result of performing limb progression during stance to move on to the next step. Walking may require a much larger decrease in this signal-to-noise ratio than standing to lead to an emerging effect in behavior due to this requirement. The healthy individuals may have received conflicting sensory information from what they predicted, resulting in a larger correction to their gait control; however, they were successfully able to make this correction, maintaining proper balance (Figure 8). Thus, to properly test if the CoP movement is used for balance control during walking, a more selective and detailed method of stimulation or task must be used.

### 4.4. Limitations

This study comes with some limitations. Firstly, it could be that inclined walking was not the best way to increase reliance on tactile feedback. A previous study found that declined and inclined walking led to a stronger effect from supra-threshold tactile stimulation on stride time [21]. However, this stimulation was provided throughout the entire gait cycle, including the swing phase. Thus, the effects found from that study may have been from the perturbing sensation of vibration during the swing, opposed to an augmentation of sensation while the foot was on the ground. Future studies should investigate if specifically stimulating the plantar surfaces during swing causes changes in gait while only stimulating during the stance phase leads to results similar to what is shown in the study.

Next, this study was performed on healthy subjects that had healthy ranges of tactile perception (Figure 5). It appears that the healthy individuals were able to easily distinguish the vibrations from the normal CoP movement. Future studies should include individuals with reduced plantar sensitivity or altered tactile perception, such as stroke survivors [15].

Finally, no direct measure of the subjects’ CoP movement was analyzed in this study. This was due to a few reasons. Firstly, with our custom insoles being placed within the shoes, this left little room for portable foot pressure-sensing insoles. Additionally, it was inconsistent to calculate the CoP from the force plates due to cross-over steps or subjects taking multiple steps on the contralateral belt. The results of the current study found no changes in balance measures from the presence of different stimulation patterns, so we do not suspect a significant change in the CoP movements. Future studies would benefit from finding a low-profile pressure-sensing insole that would not be affected by the plantar stimulation device.

## 5. Conclusions

In the current study, we investigated how different sequences of vibro-tactile stimulation altered spatiotemporal and balance measures during level and inclined walking with low vision. However, very few effects of the stimulation sequences were found. Therefore, healthy humans have the ability to adjust and reweigh, through multisensory integration and residual sensory feedback, such that gait and balance outcomes show minimal or no deficits when foot–sole tactile sensory sequences are manipulated in low-vision conditions, especially during slow walking. It is possible that the perception of pressure movements may be supplied by SA mechanoreceptor fibers that are not typically sensitive to vibrations. This work gives an indication of the flexibility and adaptability of a healthy motor control system and demonstrates a method of testing such a system with an online stimulation control software. It remains to be seen whether the specific sequence of augmented tactile stimulation could improve gait and balance metrics in individuals with sensory deficits in the foot.

## Figures and Tables

**Table 1:** The perception of pressure through monofilaments. The filaments in the different foot regions fell into healthy ranges.

Monofilament Test												
	Right (Filament)						Left (Filament)					
**Subject**	**Big Toe**	**MT1**	**MT5**	**Sole**	**Heel**	**Little Toe**	**Big Toe**	**MT1**	**MT5**	**Sole**	**Heel**	**Little toe**
**1**	4.31	2.83	4.31	3.61	4.31	~	4.31	2.83	4.31	3.61	4.31	~
**3**	3.61	4.31	3.61	3.61	4.31	~	3.61	3.61	4.31	4.31	4.31	~
**4**	4.31	2.83	4.31	4.31	4.31	4.31	2.83	4.31	2.83	2.83	4.31	4.31
**5**	3.61	3.61	3.61	3.61	4.31	~	3.61	3.61	3.61	3.61	4.31	~
**6**	2.83	4.31	3.61	4.31	3.61	3.61	3.61	3.61	4.31	3.61	4.31	4.31
**7**	4.31	4.31	4.31	4.31	4.31	4.31	4.31	4.31	4.31	4.31	4.31	4.31
**8**	3.61	2.83	4.31	2.83	3.61	3.61	3.61	2.83	4.31	2.83	3.61	3.61
**9**	4.31	3.61	4.31	3.61	4.31	4.31	4.31	3.61	3.61	3.61	4.31	4.31
**11**	3.61	3.61	4.31	4.31	3.61	4.31	2.83	3.61	4.31	4.31	4.31	3.61
**average:**	3.83	3.58	4.08	3.83	4.08	4.08	3.67	3.59	3.99	3.67	4.23	4.08
**std. dev:**	0.51	0.64	0.35	0.51	0.35	0.36	0.58	0.52	0.53	0.58	0.23	0.36
**mode:**	4.31	2.83	4.31	3.61	4.31	4.31	3.61	3.61	4.31	3.61	4.31	4.31

**Table 2:** The perception of vibration measured using a biothesiometer. All subjects were within healthy ranges.

Biothesiometer Test						
	Right (Microns)			Left (Microns)		
Subject	MT1	MT5	Heel	MT1	MT5	Heel
**1**	0.01	0.01	0.04	0.01	0.04	0.04
**3**	0.16	0.04	0.09	0.09	0.04	0.16
**4**	0.04	0.04	0.04	0.04	0.04	0.04
**5**	0.04	0.04	0.04	0.09	0.04	0.04
**6**	0.04	0.04	0.04	0.04	0.04	0.04
**7**	0.16	0.09	0.09	0.09	0.04	0.09
**8**	0.04	0.04	0.04	0.04	0.04	0.04
**9**	0.09	0.04	0.04	0.09	0.04	0.09
**11**	0.04	0.04	0.04	0.04	0.04	0.04
**average:**	0.07	0.04	0.05	0.06	0.04	0.06
**std. dev:**	0.06	0.02	0.02	0.03	0	0.04

**Table 3:** Post-test comfort scale of insoles. Comfort was reported as average on 10-point scale, with mixed responses for effect of active vibrations.

Comfort Scale			
Subject	OFF	ON	Difference
**1**	6	5	−1
**3**	5	6	1
**4**	7	6	−1
**5**	4	7	3
**6**	4	4	0
**7**	9	4.5	−4.5
**8**	5	4.5	−0.5
**9**	7	8	1
**11**	6	6	0
**average**	5.89	5.67	−0.22
**std. dev**	1.62	1.3	2.03

**Table 4:** Subject perceptions of stimulation sequences. Most subjects were unable to perceive any differences in the stimulation sequences while walking. Only one person noticed that the sequence changed, while others thought that the vibration intensity changed opposed to the pattern.

Pattern Response	
Response Examples	Response Frequency
“The stimulation felt stronger sometimes”	2
“There was a forward sequence and backward sequence”	1
“There were different patterns”	1
“No difference”	5

## Data Availability

The original contributions presented in this study are included in the article/Supplementary Materials. Further inquiries can be directed to the corresponding author (mmukherjee@unomaha.edu).

## Supplementary Materials

The following supporting information can be downloaded at https://www.mdpi.com/article/10.3390/brainsci15020138/s1. Text S1: Vibr_stim_seq_sup_surface on_gait_balance_Supp. Table S1: Results for all variables at each incline of walking and each stimulation sequence; Figure S1: Subjects were given this key to answer which tactor set was being activated during the tactor familiarization. Subjects were asked to respond with which lettered circle was activated. Figure S2: A representative example of the foot placement area calculation. Figure S3: Examples of XCoM and MoS calculations. Figure S4: Examples of failed stimulation trials. Figure S5: Box and whisker plots of step length and AP CoM velocity at heel-strike. Figure S6: Step widths four steps before and four steps after each tactile stimulation sequence, before the start and after the end of no stimulation (gray), *gait-like* stimulation (green), and *random* stimulation (red).
