# Supplementary material for: Effect of Vibro-Tactile Stimulation Sequence and Support Surface Inclination on Gait and Balance Measures
Source: Brain Sci. 2025 Jan 30;15(2):138. doi: 10.3390/brainsci15020138 (PMC11853359; doi:10.3390/brainsci15020138)
Supplement: Supplementary file 1 [file brainsci-15-00138-s001.zip › brainsci-3429252-supplementary.pdf]

Effect of Vibro-tactile Stimulation Sequence and Support Surface Inclination on Gait and Balance Measures

Christopher P. Engsberg M.S.[1], Nathaniel H. Hunt PhD[1], Steven Barlow PhD[2] & Mukul Mukherjee PhD[1]
[1] Department of Biomechanics, University of Nebraska at Omaha, Omaha, NE USA
[2] Center for Brain, Biology, and Behavior, University of Nebraska Lincoln, Lincoln, NE USA

Supplementary Figures and Tables

		NS	GS	RS
Stance Time (sec)	Level	0.843 (0.037)	0.845 (0.038)	0.849 (0.040)
	5Inc	0.866 (0.065)	0.862 (0.065)	0.859 (0.073)
	8Inc	0.863 (0.078)	0.868 (0.080)	0.860 (0.070)
Stance Length (Body Height)	Level	0.225 (0.025)	0.226 (0.025)	0.228 (0.026)
	5Inc	0.233 (0.029)	0.232 (0.025)	0.231 (0.031)
	8Inc	0.243 (0.062)	0.245 (0.063)	0.241 (0.060)
Stride Width (cm)	Level	14.20 (3.076)	14.45 (3.312)	14.39 (2.845)
	5Inc	14.86 (4.139)	14.86 (3.558)	15.06 (3.601)
	8Inc	14.86 (4.209)	14.87 (4.051)	14.98 (3.800)
Foot Placement Area (cm^2)	Level	25.09 (10.49)	23.38 (13.84)	22.65 (11.00)
	5Inc	41.17 (20.21)#	38.03 (23.48)#	35.86 (19.91)#
	8Inc	42.32 (23.65)#	39.51 (18.71)#	35.10 (12.80)#
ML MoS (cm)	Level	4.497 (1.430)	4.574 (1.483)	4.572 (1.341)
	5Inc	4.871 (1.684)	4.920 (1.612)	4.917 (1.667)

	8Inc	4.822 (1.630)	4.763 (1.609)	4.858 (1.536)
AP MoS Heel Strike (cm)	Level	4.426 (1.646)	4.401 (1.650)	4.249 (1.802)
	5Inc	1.707 (3.066)#	2.178 (3.067)#	1.960 (3.636)#
	8Inc	1.232 (3.810)#	1.291 (4.386)#	1.686 (3.541)#
AP MoS Midstance (cm)	Level	24.92 (0.772)	24.89 (0.854)	24.86 (0.831)
	5Inc	24.56 (0.708)	24.56 (0.683)	24.51 (0.774)
	8Inc	24.33 (0.614)	24.42 (0.807)	24.45 (0.623)

Table S1: Results for all variables at each incline and each stimulation sequence. # indicates a significant difference from the level incline (p<0.05). Values in parentheses represent standard deviation.

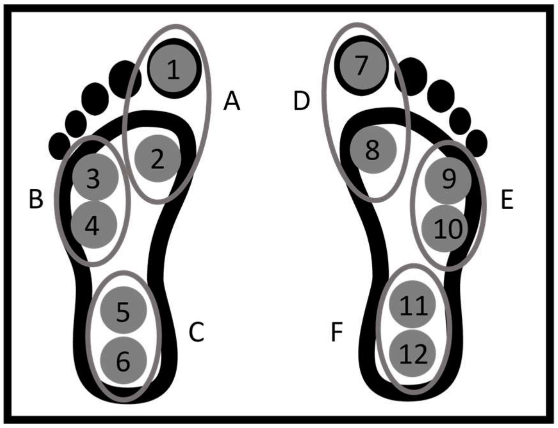

Figure S1: Subjects were given this key to answer which tactor set was being activated during the tactor familiarization. Subjects were asked to respond with which lettered circle was activated.

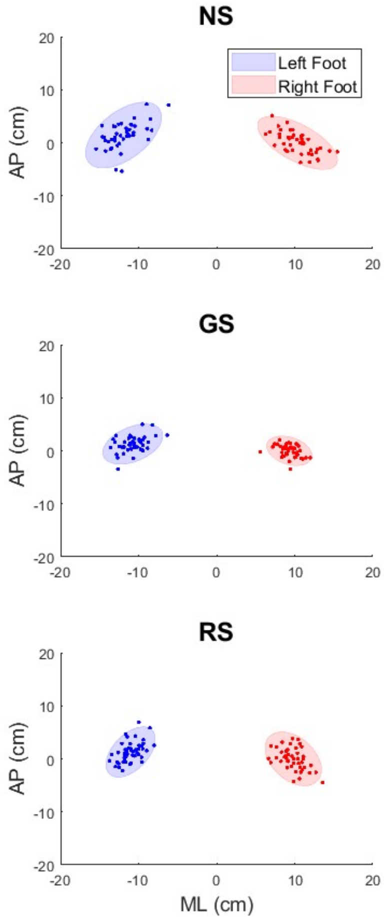

Figure S2: A representative example of the foot placement area calculation. Each colored dot represents the location of the heel, with respect to the CoM, at the moment of heel strike. Each data point was shifted down by the overall average position of both limbs' foot placements. This was performed so the y-axis would be the same for each participant and such that the orientation of both limbs' foot placements was not altered. Then, a 95% confidence interval ellipse is drawn to represent the spread of these foot placements for both feet. The area of the left and right ellipses is averaged and then compared between conditions.

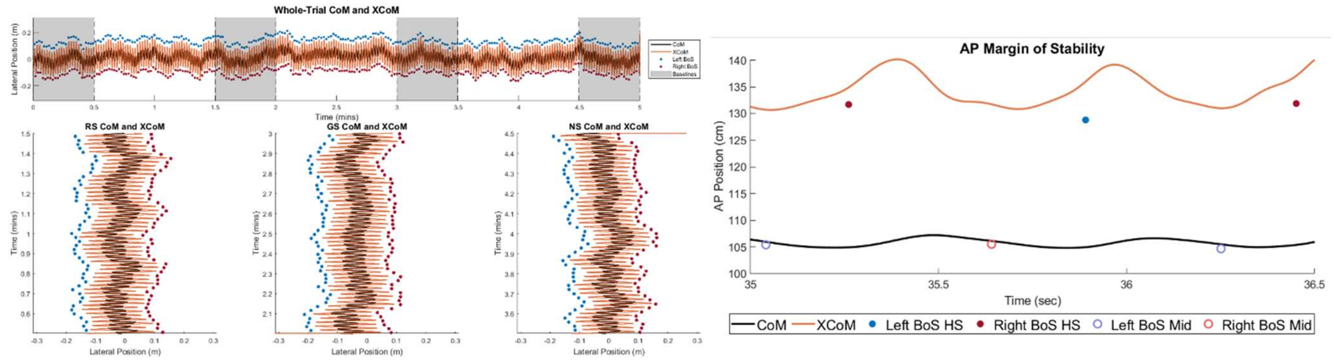

Figure S3: Examples of XCoM and MoS calculations. (**Left**) The XCoM, the CoM, and both sides' BoS are shown throughout an entire trial and are broken down to individual stimulation sequence sections. The ML MoS was determined by the distance between the peak of the XCoM and the BoS for that stance period. (**Right**) A depiction of the AP XCoM, the CoM, and both sides' BoS. The XCoM is constantly ahead of the CoM because the individual was walking at a speed of 0.8m/s in that direction. The AP MoS at heel strik was calculated as the distance between the

BoS at heel strike (solid dot) and the XCoM at the moment of heel strike (open circle of the same color). The same calculation was performed for the AP MoS at midstance but used that distance at the moment of midstance instead.

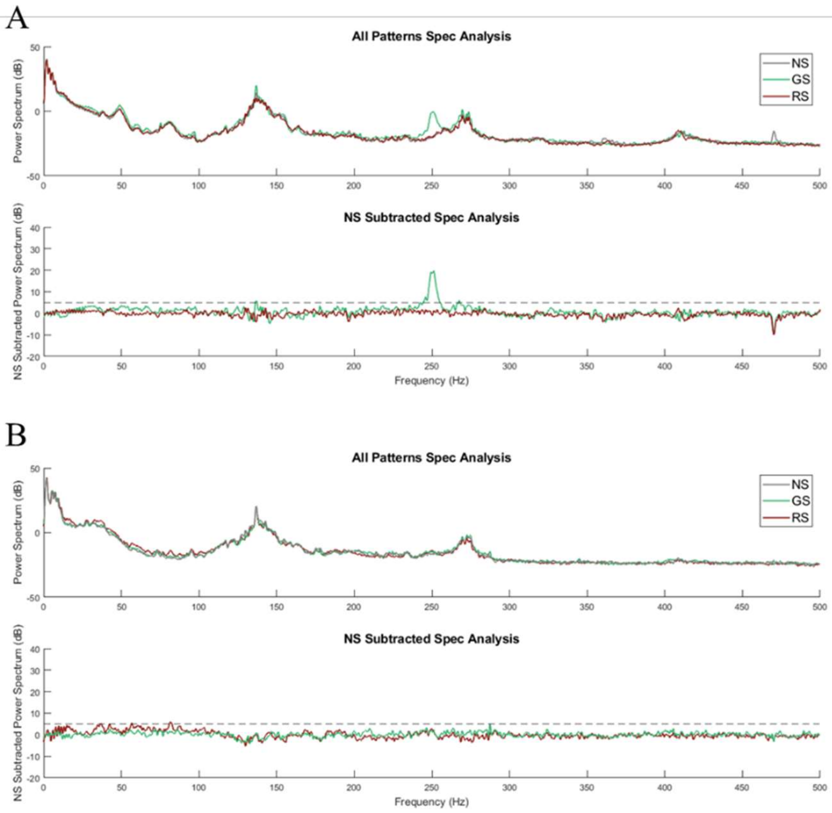

Figure S4: Examples of failed stimulation trials. (**A**) Spectral analysis of a trial where the tactor control boxes disconnected during a trial. The tactors were active at 250Hz for GS but disconnected before RS began. (**B**) A trial where tactor activation signals were sent to the tactor box but the boxes became disconnected, leading to no spectral peak at 250Hz and thus a lack of vibrations supplied to the plantar surfaces.

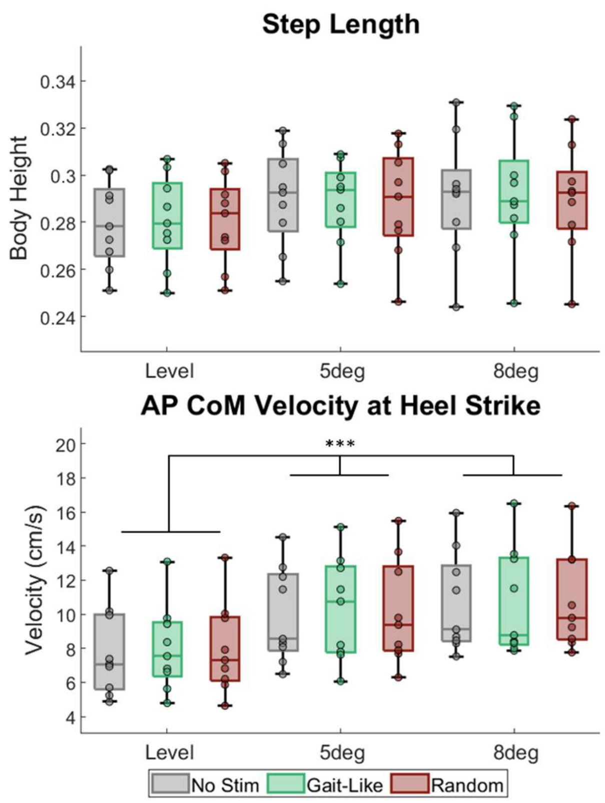

Figure S5: Box and whisker plots of step length and AP CoM velocity at heel strike. These were investigated to determine if they could be the cause of the reduced AP MoS at heel strike. The stimulation had no effect on these measures. The incline only had a significant effect on the AP CoM velocity at heel strike (F=13.303; $p<0.001$), where it significantly increased by about 2.2cm/s and 2.7cm/s when the incline went from level to 5 deg and 8 deg, respectively (*** indicates p<0.001).

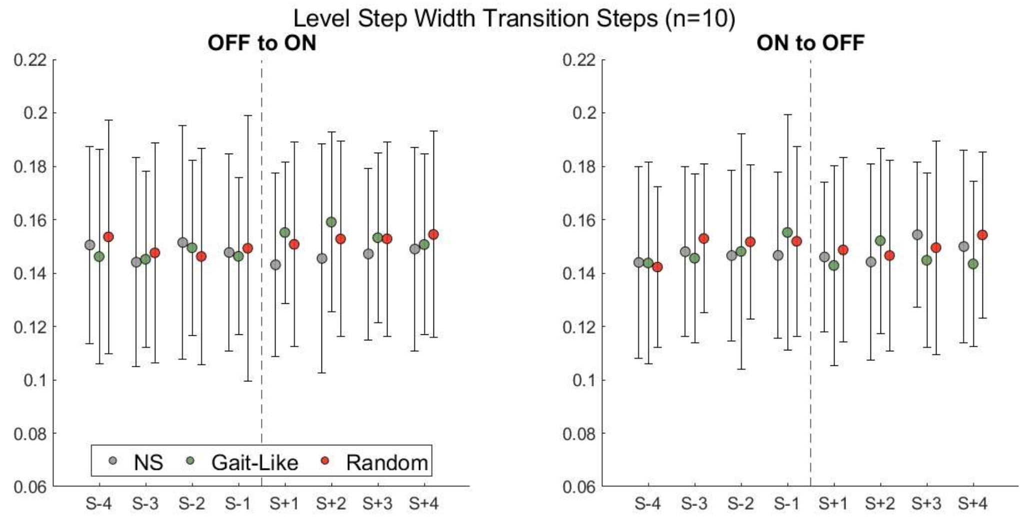

Figure S6: Step widths four steps before and four steps after each tactile stimulation sequence, before the start and after the end of no stimulation (gray), *gait-like* stimulation (green), and *random* stimulation (red). (**Left**) Average step widths across each participant before the stimulation sequence started (left of vertical dashed line) and average step widths after the stimulation sequence started (right of vertical dashed line). (**Right**) The same as **Left** except it shows

the transition steps of the stimulation pattern going from ON to OFF. Circles represent averages and brackets represent standard deviations.
